# Community-based rehabilitation implementation framework to address patellofemoral pain amongst runners in under-resourced communities: Delphi consensus

**DOI:** 10.4102/sajp.v77i1.1531

**Published:** 2021-06-22

**Authors:** Siyabonga Kunene, Nomathemba Taukobong, Serela Ramklass

**Affiliations:** 1 Department of Physiotherapy, Faculty of Health Sciences, University of the Witwatersrand, Johannesburg, South Africa; 2 School of Clinical Medicine, College of Health Sciences, University of KwaZulu-Natal, Durban, South Africa; 3 Department of Institutional Planning, Faculty of Health Sciences, Sefako Makgatho Health Sciences University, Pretoria, South Africa

**Keywords:** patellofemoral pain, community-based rehabilitation, runners, under-resourced communities, Delphi technique

## Abstract

**Background:**

Runners in under-resourced communities in parts of South Africa present with a high prevalence of patellofemoral pain (PFP), which affects their level of participation in sporting activities. Therefore, a specific rehabilitation approach is necessary to manage the PFP-related needs of these runners within their means and reach.

**Objective:**

To develop a community-based rehabilitation (CBR) implementation framework for PFP amongst runners in under-resourced communities.

**Method:**

Our study used the Delphi technique to develop an appropriate rehabilitation implementation framework for PFP in community-based settings. Sport medicine experts, involved in the treatment and rehabilitation of PFP, were recruited to participate. The Delphi process consisted of three rounds to attain consensus amongst the experts on the components and elements that could be contained in a rehabilitation implementation framework for the management of PFP. Experts rated the framework items using a five-point Likert scale.

**Results:**

A total of 19 experts participated in our study: 10 were females and 9 were males of whom 13 were aged between 36 and 55 years. Most were local experts (15) with 11–20 years of clinical experience. Four core rehabilitation implementation items were identified through the Delphi process. These were: (1) the establishment of transdisciplinary rehabilitation teams, (2) upskilling of available clinicians, their assistants and trainers, (3) implementation of a CBR programme at low-level or no-cost and (4) referral of cases to secondary or tertiary institutions for further management.

**Conclusion:**

Consensus was reached for a comprehensive CBR implementation framework aimed at addressing the specific needs of runners with PFP in under-resourced communities.

**Clinical implications:**

A further study to test the feasibility of the agreed-upon intervention is recommended.

## Introduction

Running is one of the most popular sports that is affordable to many people, especially to those living in poor socio-economic conditions with limited resources. South Africa has an active schedule of running activities that encourages many people to be physically active and provides opportunities for those interested in high-level performance games and races (e.g. the Comrades Marathon, the Two Oceans Marathon, the Olympics and the Paralympics). Unfortunately, running is accompanied by various musculoskeletal injuries including patellofemoral pain (PFP). Patellofemoral pain is considered the most common knee-overuse injury globally (Crossley et al. 2016).

A drawback to running is the high risk of injury, especially amongst runners in communities where the socio-economic conditions are substandard. Many runners in these under-resourced communities train and compete despite their injuries that are poorly managed because of limited available rehabilitation services. Patellofemoral pain results in impairments and activity limitations, which restricts participation amongst runners (Willy et al. 2019). A high prevalence of PFP amongst runners in under-resourced communities has been reported by Kunene, Ramklaas and Taukobong (2018a, 2019). They reported a PFP prevalence of 40% and identified the physical and psychological risk factors amongst runners in an under-resourced community in South Africa. In a follow-up study, Kunene et al. found that PFP adversely affected the quality of life of these runners (Kunene et al. 2018b), namely the physical, mental, social and emotional states of many runners. Runners are affected and discouraged by injuries and this might result in their withdrawal from the activity. Therefore, the management of sports injuries such as PFP becomes crucial to keep communities physically active and involved in sports.

Patellofemoral pain has been widely researched and various rehabilitation strategies are recommended. Some of these strategies include patient education about the condition and the management thereof (Esculier et al. 2018), running gait training (Willy & Davis 2013; Willy, Scholz & Davis 2012), exercise (Ferber, Kendall & Farr 2011; Neal et al. 2016), the use of orthoses (Boldt et al. 2013; Shih, Wen & Chen 2011) and multimodal rehabilitation (Esculier, Bouyer & Roy 2016). The implementation of these strategies requires resources, including skilled clinicians, equipment and appropriate healthcare facilities. Unfortunately, the lack of these resources may prevent runners from accessing good quality rehabilitative services. In a focus group interview amongst runners with PFP, the absence of affordable rehabilitation services and access to them was evident in an under-resourced community in South Africa (Kunene et al. 2020b). Similar circumstances are experienced by athletes in other inadequately resourced communities (Finch et al. 2003; Poczta & Malchrowicz 2018).

Our study thus sought to develop a suitable framework to address the rehabilitation needs of runners with PFP in under-resourced communities using Ekurhuleni in Gauteng as an example. Rehabilitation services should be available, accessible, affordable, adequate and appropriate to all people, irrespective of their age, gender, race and socio-economic status. There is a need for such services amongst runners in under-resourced South African communities (Kunene et al. 2020b). A combination of community-based rehabilitation (CBR), transdisciplinary rehabilitation (TDR) and task-shifting approaches is considered in our article as a way of addressing related needs in under-resourced communities.

A CBR approach is designed to increase access to rehabilitation services in communities where services are limited (World Health Organisation [WHO] et al. 2010). Transdisciplinary rehabilitation is a ‘specific form of interdisciplinary care in which boundaries between and beyond disciplines are transcended and knowledge and perspectives from different scientific disciplines and non-scientific sources are integrated’ (Flinterman et al. 2001). The TDR approach addresses the challenge of scarce rehabilitation skills, which is acute in low-income countries, including South Africa (Rasool & Both 2011). Task-shifting is defined as ‘delegating tasks to existing or new cadres with either less training or narrowly tailored training’ (Fulton et al. 2011). All these approaches have not been used in combination to address the rehabilitation needs of runners including those with PFP, especially in under-resourced communities.

## Methods

A Delphi method was used to develop and prioritise the content of a rehabilitation implementation framework for rehabilitation of running-related PFP injuries in under-resourced communities. This method seeks consensus amongst a group of experts and is a useful tool highly commended in the development of effective interventions (Giannarou & Zervas 2014).

A panel of experienced sports clinicians was headhunted and recruited by the first author to participate in our study. The first author included experts from various parts of South Africa and clinicians who were experienced in working with runners in marginalised communities. Experts from the United Kingdom who had a similar experience were also included. The experts included sports physicians, physiotherapists, sports therapists, biokineticists, podiatrists, dieticians and psychologists. The participants had to have at least 5 years of experience in the treatment and rehabilitation of running-related injuries including PFP. Electronic mails were used as a form of communication between the first author and the participants. All participants were provided with the relevant study information, an invitation to participate and a consent form.

### Procedure

Consent was obtained from all participants prior to the Delphi process. Initially, participants’ demographic data were collected with the aid of a questionnaire developed by the first author. The data collected included gender, age, profession, current position and years of professional experience in the treatment and rehabilitation of PFP.

#### The Delphi process included three rounds:

**FIGURE 1:**
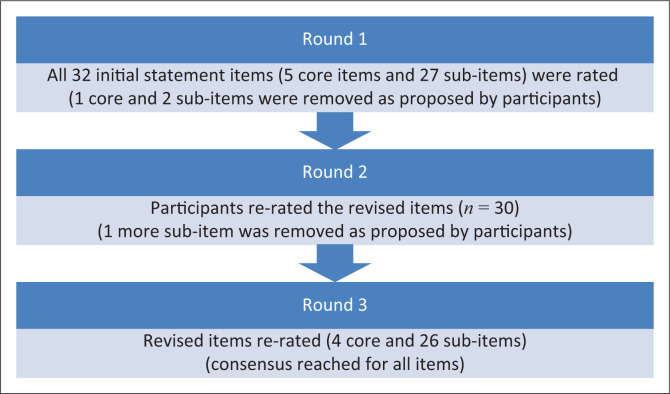
Flow diagram to show the Delphi three survey rounds.

Round 1: Evidence from relevant articles was used to generate 32 statement items (five core items and 27 sub-items) on how rehabilitation programmes for runners with PFP can be implemented in under-resourced communities. In addition, the outcome from studies on the nature of PFP, runners’ needs in under-resourced communities and current rehabilitation strategies were also used (Kunene et al. 2018a, 2018b, 2019, 2020a, 2020b). The 32 preliminary statement items were converted into a questionnaire and pilot tested amongst three sports medicine academics, one sports physician and one clinical physiotherapist. The three survey rounds of our Delphi process are illustrated (See Figure 1). Following the pilot study, the questionnaire was disseminated to the 19 members of the Delphi panel via emails (in a Microsoft Word document). Participants were asked to review and rate each item using a five-point Likert scale, ranging from 0 to 4 (4 = strongly agree, 3 = agree, 2 = disagree, 1 = strongly disagree and 0 = neutral). A space for comments was provided in the questionnaire. Three statement items (one core item and two sub-items) from the 32 were removed as proposed by participants.

Round 2: In this round, the first author analysed the responses from round 1. Scores of each participant were established and items that did not reach the pre-determined conceptual score of at least three out of four were excluded. This score indicated that a participant agreed or strongly agreed. Similar studies have used Likert scales to obtain consensus from Delphi participants (Diamond et al. 2014; Henderson & Rubin 2012; Slade et al. 2014; Vogel et al. 2019). In this round, the list of items was reviewed taking into consideration the suggestions from the experts. A revised list of 30 statement items and round 1 scores was shared with the same panel via email. Instructions from round 1 were given again for this round. Electronic mail reminders were also sent to participants within 3 weeks to encourage responses. One more sub-item statement item from the revised 30 items was removed as proposed by participants.

Round 3: The first author analysed round 2 responses and excluded the items that scored less than three out of four. A final list of 29 items was then prepared and sent back to participants for the final rating. No more statement items were removed, meaning that consensus had been reached.

Framework draft: After consensus was reached in round 3, the rehabilitation implementation framework was compiled in a detailed Microsoft Word document for final comments.

### Data analysis

Descriptive statistics were used to present the demographic profile of the participants and their responses to the Delphi questions. The acceptable level of consensus was set at >70% of the Delphi participants who agreed or strongly agreed in round 3. Previous Delphi studies have accepted this level of consensus (Diamond et al. 2014; Henderson & Rubin 2012; Slade et al. 2014; Vogel et al. 2019).

### Ethical considerations

Ethical approval was obtained from the Biomedical Research Ethics Committee of the University of KwaZulu-Natal (Ethical clearance number: BFC377/15).

## Results

### Participants

Attributes of the Delphi participants are outlined (Table 1). The study recruited 19 participants who were healthcare practitioners and experts in the field of sports medicine. A total of 22 potential participants were invited; however, 19 accepted the invitation and thus participated. Most of the participants were females (*n* = 10) with ages ranging between 36 and 55 years (*n* = 13). The participants’ mean age was 46 years. A total of 15 participants were practising their profession in South Africa and 4 were based in the United Kingdom. The majority (*n* = 10) had 11–20 years of clinical experience. The mean for participants’ clinical experience was 16 years.

**TABLE 1:** Demographic profile of participants (*n* = 19).

Characteristics	Categories	*n*
Gender	Male	9
	Female	10
Age	18–35	4
	36–55	13
	> 55	2
Geographical location	Local	15
	UK	4
Profession	Physician	3
	Physiotherapist	6
	Sports therapist	2
	Biokineticist	2
	Podiatrist	2
	Dietician	2
	Psychologist	2
Years of clinical experience	5–10	3
	11–20	10
	> 20	6

**FIGURE 2:**
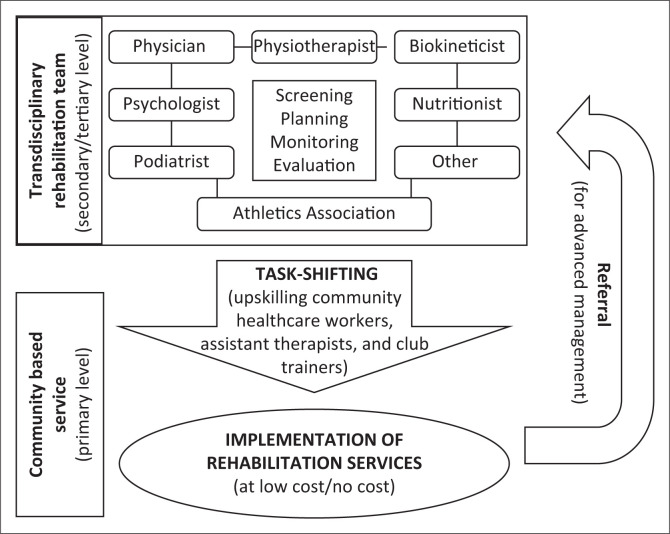
Proposed framework for injury rehabilitation amongst runners in under-resourced communities.

The Delphi process yielded four core items of the CBR implementation framework for runners with PFP in under-resourced communities. These were: (1) the establishment of a TDR team (with an average score of 4 out of 4), (2) upskilling of the less skilled (task-shifting) (with an average score of 3 out of 4), (3) implementation of a CBR programme at low-level or no-cost (with an average score of 3 out of 4) and (4) referral of cases to secondary or tertiary institutions for further management (with an average score of 4 out of 4). The framework is outlined in Figure 2.

### Establishment of transdisciplinary rehabilitation teams

All the 19 participants (100%) agreed that a transdisciplinary rehabilitation team be developed at either secondary or tertiary healthcare centres or at academic institutions that provide outreach to under-resourced communities. The representatives from the athletic federations who serve community clubs will also be included as rehabilitation team members. This rehabilitation team will be responsible for injury screening, planning, monitoring and evaluating the rehabilitation services offered for runners in under-resourced communities.

### Upskilling of the less skilled

Seventeen (17) out of 19 (90%) participants agreed to the process of identifying and upskilling less-skilled personnel in the local community. As a result of the scarcity of sports rehabilitation services in South African low socio-economic communities, cross-training or the upward skills development of the available community healthcare workers, assistant therapists and club trainers was proposed for the effective implementation of the rehabilitation programmes. The training was proposed to be carried out by the established TDR teams.

### Implementation of a community-based rehabilitation programme at low-level or no-cost

Eighteen (18) out of 19 (95%) agreed to the implementation of CBR programmes at a low-level or no-cost. Pro Deo services were suggested to be the appropriate method to make services freely available for runners who cannot afford them. These services were proposed to be offered by the upskilled community healthcare workers, assistant therapists and club trainers. Rehabilitation programmes would be structured to encourage clubs to take ownership of their exercise rehabilitation programmes with less involvement of a clinician. Club trainers would have to be fully involved to ensure that the runners comply with their prescribed exercise rehabilitation programmes.

### Referral of cases to secondary or tertiary institutions for further management

All 19 participants (100%) agreed to the referral of cases for further injury management at a secondary and tertiary healthcare facility. The upskilled personnel working with runners in a local community would be responsible for identifying runners whose injuries are not improving with the implementation of the exercise rehabilitation programme and for referring them for further management. Clinicians (members of the established TDR team) would then be expected to further manage the referred cases. A low or no-cost service is still proposed even at this level of intervention.

## Discussion

Studies have shown that runners in under-resourced communities have a high prevalence of poorly managed PFP injuries because of inaccessible, unaffordable, unavailable and inadequate rehabilitation services (Kunene et al. 2018a, 2018b, 2019, 2020a, 2020b). South Africa faces the challenge of scarce healthcare resources, especially in low socio-economic communities, which makes it difficult to fully address the health-related needs of people (Rasool & Both 2011). This challenge makes it difficult for runners to receive the necessary healthcare management for injuries sustained whilst engaged in running-related activities. Our study proposes a rehabilitation implementation framework, which is a community-based approach with incorporated transdisciplinary and task-shifting models to manage PFP injuries in these communities. The needs of runners in these communities are diverse, therefore a suitable rehabilitation framework is necessary to address them. The proposed framework comprises four core items: (1) the establishment of TDR teams, (2) upskilling of available community healthcare workers, assistant therapists and club trainers, (3) implementation of a CBR programme at low-level or no-cost and (4) referral of cases to secondary or tertiary Institutions for further management. The rehabilitation framework is not only applicable to the rehabilitation of PFP but could also be adapted to the rehabilitation of general running injuries amongst athletes in under-resourced communities, for example, rural, townships and semi-urban communities.

The developed rehabilitation framework has combined and integrated concepts from the transdisciplinary-rehabilitation model, CBR strategy and task-shifting concept (Dawad & Jobson 2011; Mauk 2012). The Delphi experts proposed that the rehabilitation team be transdisciplinary in nature, meaning that the available clinicians at the community healthcare facility (e.g. secondary or tertiary hospital) will work together but with a need for their work boundaries to be transcended and integrated (Flinterman et al. 2001). Transdisciplinary rehabilitation models have been used in various settings, especially in long-term acute care (Reilly 2001). However, this model has not been explored in the context of running injury rehabilitation. The experts in our Delphi study agreed that the first step in managing running-related injuries in under-resourced communities is to establish a TDR team. The team should be comprised of various sports clinicians, who should volunteer their services and work together as a rehabilitation management team for under-resourced communities. The said TDR teams should also be responsible for injury screening, planning, implementing, monitoring and evaluating rehabilitation programmes. The approach promises to promote the working together of rehabilitation clinicians and the sharing of roles across disciplinary boundaries to make services available, accessible, efficient and cost-effective (ed. Davies 2007; eds. Johnson et al. 1994; King et al. 2009).

The TDR approach provides the opportunity for a cross-trained, multiskilled and well-equipped team to plan, implement, monitor and evaluate a comprehensive rehabilitation programme where resources are scarce (Mauk 2012). Because of the scarcity of sports clinicians in communities where poor socio-economic conditions prevail, the available clinicians at secondary or tertiary institutions should be multiskilled or cross-trained beyond their disciplinary boundaries. Consequently, extra knowledge and skills will be borrowed from other disciplines to implement a rehabilitation programme for complex PFP injuries. Forming successful rehabilitation teams may be a challenge in poorly resourced communities owing to the lack of formal structures, support and trained professionals (Finch et al. 2003). The establishment and sustainability of the proposed transdisciplinary community rehabilitation teams are dependent on the cooperation of the relevant stakeholders. Therefore, it is critical that the Department of Sports and Recreation (2012) prioritises the rehabilitation of injuries and provides support for the establishment of rehabilitation teams to rehabilitate athletes in disadvantaged communities. Local athletic associations may need to be supported with adequate resources to offer rehabilitative services to the runners. The Department of Health should also play a role in facilitating the establishment of rehabilitation teams in communities and in providing healthcare facilities conducive to meeting the needs related to running injuries.

For the rehabilitation services to reach every runner, further training and upskilling of less-skilled personnel available in the local communities is necessary. Community healthcare workers, therapist assistants or technicians and club trainers may be trained and upskilled to implement rehabilitation programmes in their local communities. Established rehabilitation teams at a secondary or tertiary level might not do all the work on their own. Therefore, our study proposes that the established rehabilitation teams at secondary or tertiary institutions be responsible for the training and upskilling of the less skilled so that the work can be implemented in the communities. For any rehabilitation programme to succeed, the club trainers will also need to be provided with the relevant knowledge and skills in running injuries including PFP injury prevention and rehabilitation. Trainers are key role-players in the rehabilitation of running-related PFP injury because of the influence they have on athletes. In order to assist with prevention and management of PFP injuries, trainers should be given the appropriate knowledge and skills. They are a good resource to also provide emotional, mental, social, material and informational support to runners (Johnson et al. 2011; Podlog & Eklund 2007). Furthermore, they would be shown the skills for adapting their training or coaching schedules to accommodate those runners recovering from PFP injuries.

Unfortunately, most sports trainers in poor communities do not have the necessary knowledge and skills to prevent and rehabilitate running-related PFP injuries. They have not gone through the proper training for effective coaching because there is a lack of appropriate resources and they tend to rely on their own limited experience. A well-empowered sports trainer can plan and implement safety training programmes that do not put the runner at risk of PFP injuries. Therefore, the relationship amongst a clinician, coach and runner must be strengthened to ensure that PFP injuries are effectively prevented and the runners are fully rehabilitated (Johnson et al. 2011; Podlog & Eklund 2007). Such sound relationships amongst the parties concerned will promote good communication and improve trust and, also, they should lead to an understanding of the injury and rehabilitative process. The proposal made in our study of training and skilling less-specialised people in the community is a concept borrowed from the task-shifting model where there is a delegation of task from highly specialised to less-specialised people to tackle a health issue head-on (WHO 2008). This approach has been successfully implemented in various contexts outside the sports arena. Dawad and Jobson (2011) successfully implemented a community-based programme for the treatment of human immunodeficiency virus (HIV) patients in rural communities using multiskilled mid-level workers who had been effectively trained. The task-shifting model is proposed as an appropriate model to assist in CBR programmes, especially where there is a paucity of healthcare professionals. To deal with problems of limited sports rehabilitation resources in low socio-economic communities, training and upskilling of available and willing community service therapists, health technicians and sports trainers are critical.

A framework proposed by the National Department of Health (DOH) is synergistic to that proposed in our study. The National DOH (2016) published a framework and strategic document on disability and rehabilitation services in South Africa that proposed the establishment of CBR programmes in under-resourced communities. Owing to the scarcity of healthcare personnel, the DOH has planned to train community health workers to offer rehabilitation services in their allocated places of work.

Prevention and rehabilitation programmes for PFP consist mostly of exercises that can be taught and delegated (taking into consideration the scope of practice of each person according to the Health Professionals Council of South Africa) to a less-specialised person in the community to implement, for example, healthcare workers or sports trainers. The exercise rehabilitation programme for PFP may include strengthening exercise of the proximal lower limb muscles including muscles controlling the hip (e.g. external rotators and hip abductors) and knee (e.g. vastus medialis oblique) (Brukner & Khan 2017; Halabchi, Mazaheri & Seif-Barghi 2013). Core stability exercises also form part of the programme. Stretching of the shortened iliotibial band (ITB), hamstrings, iliopsoas and calf muscles are also included in the PFP exercise programme. Education, gait retraining, training loading, foot orthosis and psychological rehabilitation are other important strategies included in addressing PFP amongst runners (Kunene et al. 2020a; Maclachlan et al. 2017).

The CBR rehabilitation implementation framework proposed in our study suggests that a rehabilitation programme for PFP be implemented in under-resourced communities at a low-level or no-cost to ensure that these services are affordable to the local communities. Professionals volunteering to provide rehabilitation services in these communities may need to consider pro Deo services. With the South African government introducing the National Health Insurance (NHI) system, this framework will be more applicable. The NHI programme is aimed at providing healthcare services that are equally available, accessible, affordable, adequate and appropriate for all South African citizens (National Health Department 2017).

The last step featured in the proposed framework deals with the referral of runners with PFP injuries to secondary or tertiary healthcare facilities for further management of their injuries. In cases where runners require advanced interventions (e.g. foot orthosis, physiological rehabilitation, medical treatment, radiological services, surgical management, etc.), which cannot be implemented in the local community, a referral will have to be made for such runners to be attended to at the next level of care, secondary or tertiary institution. We recommend that specialised sports clinics be established at secondary and tertiary levels (local healthcare or an academic institution), where athletes from under-resourced communities may be provided with advanced and specialised services once they have been referred from a primary healthcare level. It is advisable that the government, through the DOH, or the Department of Sports and Recreation (2012) subsidise these runners so that they can benefit from these rehabilitation services and also the aforementioned can provide the necessary resources and facilities to the sports clinics. The private sector may need to come on board in providing support or offering affordable rehabilitation services to these runners in poor communities.

## Conclusion

Our study established a consensus-based CBR implementation framework for athletes in under-resourced communities. The framework proposes the establishment of a TDR team to screen for PFP injuries and plan, implement, monitor and evaluate rehabilitation programmes for runners in under-resourced communities. The rehabilitation team will also be responsible for transferring their knowledge and skills to less-skilled personnel to ensure that the whole community is catered for (a task-shifting approach). Community rehabilitation services are proposed at a low-level or no-cost to make services equally available and affordable for runners. The last step of the proposed framework deals with the referral of cases for further management at secondary or tertiary institutions. For the proposed framework to be rolled out to the targeted communities, a pilot study is recommended to determine its feasibility. The implementation and evaluation of the proposed intervention will then need to be followed.
